# Editorial: Improving memory deficits in Alzheimer's disease

**DOI:** 10.3389/fnagi.2022.1066598

**Published:** 2022-11-21

**Authors:** Fushun Wang, Ralf J. Braun, Valentina Echeverria, Shijun Xu

**Affiliations:** ^1^Institute of Brain and Psychological Sciences, Sichuan Normal University, Chengdu, China; ^2^Research Area Neurodegenerative Diseases, Center for Biosciences, Faculty of Medicine/Dental Medicine, Danube Private University, Krems an der Donau, Austria; ^3^Bay Pines VA Healthcare System, Veterans Health Administration, United States Department of Veterans Affairs Bay Pines, Bay Pines, FL, United States; ^4^Facultad de Medicina, Universidad San Sebastián, Concepción, Chile; ^5^School of Pharmacy, Chengdu University of Traditional Chinese Medicine, Chengdu, China

**Keywords:** Alzheimer's disease (AD), astrocyte, glymphatic system, memory, stress, cognitive deficit

Alzheimer's disease (AD) is one of the most prevalent dementia causes, and accounts for > 80% of cases in the elderly. In addition, the incidence rate of AD is increasing, especially in people that suffered COVID-19. It is estimated that there will be over 150 million people with dementia across the world in 2050. This will put an immense pressure on social economic and healthcare and decrease the quality of life of the global population. The causes of the AD are still far from clear, despite many advances in recent years showing a prominent role of inflammation and the immune system. The most prominent symptom of AD is a progressive deterioration of cognitive functions, which includes working memory and long-term episodic memory. These impairments result in a wide range of devastating problems ranging from difficulties in simple daily lives to a complete loss of independent living and premature death.

Memory is affected by many factors, including both cognitive and emotional factors. Many studies have suggested that emotion arousal can enhance memory in both normal adults and AD patients (Nashiro and Mather, 2011; Kumfor et al., 2014). Previous studies in our lab have found that monoamine neuromodulators are the major substances for emotional arousal (Gu et al., 2018). And many studies showed that emotion related memory performance, central monoaminergic function and sympathetic nerve activity were lower in patients with dementia of the Alzheimer type (Corona et al., 1989). In addition, the monoamines can affect the function of astrocytes as well as the glymphatic system (Gu et al., 2019). It is found that chronic stress related monoamine neuromodulators such as norepinephrine can impair the function of glymphatic system and lead to tau accumulation and thus AD, while sleep can help the clearance function of the glymphatic system (Nedergaard and Goldman, 2020). In addition, the control of the cholinergic system with modulators of the nicotinic acetylcholine receptors with nicotine derivatives such as Cotinine to reduce neuroinflammation and control stress resilience are new avenues explored (Echeverria et al., 2016).

Glymphatic system is modulated by stress and related monoamine neuromodulators, such as norepinephrine, dopamine and serotonin as well as choline, and affects the clearance of Aβ and tau protein (Iliff et al., 2015). In contrast, impairment of glymphatic system might be the major reason for AD (Nedergaard and Goldman, 2020). The glymphatic system is a recently found network that functions to clean up the protein metabolite and cell debris in the extracellular milieu, which plays a pivot role controlling inflammation and maintenance of brain homeostasis (Rasmussen et al., 2018). The structure of the glymphatic system has been recently defined with immunofluorescence staining of the meninges in the whole brain of mice (Louveau et al., 2015).

The discovery of the cerebral glymphatic system has provided a revolutionary perspective to elucidate the pathophysiological mechanisms of many neurodegenerative disorders (Mestre et al., 2020). The glymphatic system is regarded as a pivot supplementary system for blood circulation, which helps maintain homeostasis by removing large molecular substances produced by cell death or metabolism, and inducing immune reactions (Wen et al., 2022). Indeed, it is recently found that the impaired protein degradation and accumulation of inflammatory molecules as well as accumulation of ROS (reactive oxygen species) and autophagy might be the mechanisms of cognitive deterioration in the progression of AD (Li et al., 2021).

However, the treatment is far from effective, except that early prevention can alleviate the progressive deterioration of serious cognitive abilities. Searching for effective therapeutic approaches are critically important for the early diagnosed patients. The aim of this Research Topic is to collect recent studies on early diagnosis of AD by measuring memory performance and novel therapeutic approaches targeted at preventing memory loss in AD. As the brain abnormalities underlying memory deficits in AD are complex and multifactorial, we encouraged submissions investigating a wide range of therapeutic targets in patients and animal models. Our topic includes 12 papers describing new therapeutic approaches as follows.

In the experimental paper titled “*Attenuation of HECT-E3 ligase expression rescued memory deficits in 3xTg-AD mice*,” Suresh et al. studied the role of HECT-E3 ligases pathways in the context of AD pathogenesis by measuring the expressions of HECT-E3 ligases and their effects on inflammasome in autophagy. They found that M01 treatment reversed the HECT-E3 ligases induced working memory deficits of 3xTg-AD mice in T-maze and Morris water maze. In addition, the expression of the NLRP3 inflammasome protein were decreased together with the improved memory performance. They suggested that modulation of HECT-E3 ligase expression might be a strategy to treat early memory deficits in AD by decreasing NLRP3 activity and increasing the autophagy pathway.

In the experimental study, Zhao et al. introduced another paper titled “*Dihydroartemisinin Ameliorates Learning and Memory in Alzheimer's disease through Promoting Autophagosome-Lysosome Fusion and Autolysosomal Degradation for A*β *Clearance*.” In the paper, the authors investigated the effects of Dihydroartemisinin (DHA) on autophagy, which plays a pivot role in removing the damaged cell components, as well as the removal of toxic protein aggregates intracellularly and extracellularly to prevent AD. In this study, the authors found that DHA treatment can promote the clearance of Aβ fibrils, improve autophagy dysfunction, and have a multi-target effect on the neuropathological process, memory, and cognitive deficits of AD.

Jia et al. from University of Toronto reported one experimental paper, titled “*Inhibition of Rac1 in ventral hippocampal excitatory neurons improves social recognition memory and synaptic plasticity*.” In the paper, the authors probed into the effects of Rac1 in AD, which is critically involved in the regulation of the actin cytoskeleton, neuronal structure, synaptic plasticity and memory, and they found that the reduction of Rac1 hyperactivity in ventral hippocampal excitatory neurons improves social recognition memory in APP/PS1 mice.

In the experimental paper titled, “*Dihuang-Yinzi Alleviates Cognition Deficits via Targeting Energy-related Metabolism in an Alzheimer Mouse model as Demonstrated by Integration of Metabolomics and Network Pharmacology*,” Han et al. introduced a kind of Chinese herb, named Dihuang-Yinzi (DHYZ), which has been used to treat AD and other neurodegenerative diseases for centuries. It is suggested energy metabolism disturbance and the consequent ROS overproduction play a key and pathogenic role in the onset and progression of Alzheimer's disease (AD). In the present study, the authors provided a novel, comprehensive and systematic insight into the therapeutic efficacy of DHYZ against AD *via* ameliorating energy-related metabolism.

In the experimental study, Shi et al. investigated the phosphodiesterase 4 (PDE4) dependent cAMP signaling in cognitive impairment associated with AD. They introduced the paper titled “*Phosphodiesterase-4D knockdown in the prefrontal cortex alleviates memory deficits and synaptic failure in mouse model of Alzheimer's disease*,” which demonstrated that RNA interference (iRNA)-mediated PDE4D knockdown in the prefrontal cortex ameliorated memory loss, which is associated with synaptic failure in AD by its antioxidant, antiapoptotic and neuroprotective properties.

Ryou et al. reported an experimental study in the paper titled “*Intermittent Hypoxia Training Prevents Deficient Learning-Memory Behavior in Mice Modeling Alzheimer's disease: A Pilot Study*.” The authors used a mouse model of AD to investigate the effects of normobaric intermittent hypoxia training (IHT) on learning-memory behavior, and the results suggested that IHT diminishes Aβ accumulation in cerebral cortex and hippocampus, and increases the level of cerebrocortical brain-derived neurotrophic factor (BDNF) in a mouse model of AD-like pathology. They concluded that moderate normobaric IHT prevented spatial learning-memory decline and restored cerebrocortical BDNF contents despite ongoing Aβ accumulation in 3xTg-AD mice. This finding is in agreement with previous studies showing an amelioration of cognitive abilities and a decrease of neuroinflammatory factors in the brain of AD mice treated with cRaf inhibitors (Burgess and Echeverria, 2010).

In the experimental study, Park et al. reported their studies about neurophysiological changes after a 24-week multi-domain intervention program in the paper titled “*Functional Brain Changes Using Electroencephalography after a 24-week Multidomain Intervention Program to Prevent Dementia*.” They showed positive biological changes, including increased functional connectivity and higher global efficiency on QEEG after a multidomain intervention program.

Brain-gut axis has recently been found to be involved in many neurodegenerative diseases (Xu et al., 2022). In the paper titled “*Effects of Tempeh Probiotics on Elderly with Cognitive Impairment*,” Handajani et al. showed that probiotic supplement-based therapies that alter the composition of gut microbiota can help prevent cognitive decline.

Blume et al. from German introduced another paper titled “*Chronic PPAR*γ *Stimulation Shifts Amyloidosis to Higher Fibrillarity but Improves Cognition*.” In the paper, the authors performed longitudinal β-amyloid positron emission tomography (Aβ-PET) imaging while treated the Aβ model mice with peroxisome proliferator-activated receptor gamma (PPARγ) agonist pioglitazone. And they found that the treatment rescued from increases of the Aβ-PET signal while promoting spatial learning and preservation of synaptic density.

In the experimental study, Zheng et al. designed a study to test the feasibility of using hybrid convolutional neural networks and long-short term memory (CNN-LSTM) modeling in the treatment of AD. In the report titled “*Predicted cognitive conversion in guiding early decision-tailoring on patients with cognitive impairment*,” the authors suggested that the utilization of deep learning modeling and featured longitudinal information might be a good therapeutic strategy for AD.

In another study, Yamashita et al. reported a paper titled “*Long-term Effect of Acetylcholinesterase Inhibitors on the Dorsal Attention Network of Alzheimer's disease Patients: A Pilot Study Using Resting-state Functional Magnetic Resonance Imaging*.” In the paper, the authors used resting-state functional magnetic resonance imaging (rs-fMRI) to probe into the physiological dynamics of functionally connected brain networks in AD. The study found that the negative correlation between the frontal cortex and the dorsal attention network may be a biomarker of the therapeutic effect of AChEIs for AD.

In another paper titled “*Precision repetitive transcranial magnetic stimulation over the left parietal cortex improves memory in Alzheimer's disease: A randomized, double-blind, sham-controlled study*,” Jia et al. introduced a study about the effect of precision repetitive transcranial magnetic stimulation (rTMS) over the left parietal cortex on the memory and cognitive function in Alzheimer's disease (AD). They found that the target site of left parietal cortex can improve AD patients' cognitive function, especially memory, providing a potential therapy (Burgess and Echeverria, 2010).

Altogether, this topic highlight potential new therapies targeting a broad range of brain processes at functional and molecular level to treat AD. We hope that this Research Topic will inspire scientist worldwide to advance basic and clinical research to find effective therapies to treat AD.

## Author contributions

FW wrote the paper. RB, VE, and SX helped with the revision. VE helped with manuscript writing. All authors contributed to the article and approved the submitted version.

## Funding

This study was supported by the grants from the project supported by National Natural Science Foundation of China, China (No. 82171392) and Fondecyt, ANID 1190264 (VE).

## Conflict of interest

The authors declare that the research was conducted in the absence of any commercial or financial relationships that could be construed as a potential conflict of interest.

## Publisher's note

All claims expressed in this article are solely those of the authors and do not necessarily represent those of their affiliated organizations, or those of the publisher, the editors and the reviewers. Any product that may be evaluated in this article, or claim that may be made by its manufacturer, is not guaranteed or endorsed by the publisher.
